# Toward objective biomarkers in psychiatric rehabilitation: detecting the “Berger Effect” with a sheet-type EEG device

**DOI:** 10.3389/fpsyt.2025.1503715

**Published:** 2025-05-06

**Authors:** China Shiroma, Keita Ueno, Masaya Ueda, Takao Inoue, Masahiro Hata, Shun Takahashi, Keigo Shiraiwa, Junya Orui, Fumie Tazaki, Yasuo Naito, Ryouhei Ishii

**Affiliations:** ^1^ Department of Occupational Therapy, Graduate School of Rehabilitation Science, Osaka Metropolitan University, Osaka, Japan; ^2^ Department of Occupational Therapy, Morinomiya University of Medical Sciences, Osaka, Japan; ^3^ Department of Psychiatry, Osaka University Graduate School of Medicine, Osaka, Japan; ^4^ Clinical Research and Education Center, Asakayama General Hospital, Osaka, Japan; ^5^ Department of Neuropsychiatry, Wakayama Medical University, Wakayama, Japan; ^6^ Department of Rehabilitation, Osaka Kawasaki Rehabilitation University, Osaka, Japan; ^7^ Department of Health Science, Osaka Health Science University, Osaka, Japan; ^8^ Graduate School of Landscape Design and Management, University of Hyogo, Hyogo, Japan

**Keywords:** Berger effect, EEG, sheet-type EEG device, HARU-1, alpha wave

## Abstract

For individuals undergoing psychiatric rehabilitation, there is an urgent need to establish an objective biomarker of brain activity without invasive or behavioral restrictions. To investigate an objective index for potential psychiatric rehabilitation assessment and intervention strategies in the future, we tried to detect the “Berger effect”, the alpha EEG reactivity to eyes opening, and to verify the suitability of a sheet-type electroencephalography (EEG) device, HARU-1. We measured EEG of 20 healthy young to middle-aged subjects using HARU-1 from the forehead area during resting closed-eye and open-eye conditions. Fast Fourier transform and time-frequency analysis were then performed on the data from all subjects in the resting closed-eye and resting open-eye conditions. Statistical analysis found that the reference electrode derivation method showed a significant increase in alpha waves and a significant decrease in beta and gamma waves in the three forehead channels of Fp1, Fp2 and AFz during the resting closed eye condition compared to the resting open eye condition. In the bipolar derivation method, all Fp1-AFz, Fp2-Fp1 and AFz-Fp2 showed a significant decrease in the β and γ bands at approximately 13–50 Hz. These results demonstrated that HARU-1 accurately measured the “Berger effect” in the forehead region, suggesting that HARU-1 may be useful in detecting objective biomarkers of brain activity during psychiatric rehabilitation.

## 1 Introduction

The introduction of the EEG was an important milestone in the progress of the neurosciences, neurology and psychiatry. Hans Berger, the pioneering German psychiatrist, is credited with inventing human electroencephalography (EEG), a term he first named. On 6 July 1924, he performed the first human EEG recording and unexpectedly observed that when the eyes were open, EEG oscillations in the alpha range (8–13 Hz) decreased in amplitude or ceased altogether (1, 2). The “Berger effect” is also called “alpha blocking”, a name that describes this phenomenon (3).

Although there are currently a number of non-invasive methods for recording brain activity, including positron emission tomography (PET), single photon emission computed tomography (SPECT), functional magnetic resonance imaging (fMRI) and other non-invasive methods, EEG is still widely used in neuroscience and clinical medicine because it is a cheap, simple and non-invasive way to measure the electrical activity of neurons (4).

Although the posterior alpha rhythm is the best-known physiological phenomenon in human electroencephalography (EEG) since its discovery by Berger (1), its genesis and functional significance is still widely discussed and not fully understood. The posterior alpha rhythm is a distinctive feature of the normal brain in the waking state, consisting of oscillations within the 8–15 Hz frequency range over posterior cortical regions which subserve visual information processing (5). This activity is observed when subjects are with their eyes-closed, under conditions of physical relaxation and relative mental inactivity (2), and shows a typical reactivity to eyes-opening and therefore to visual stimuli (i.e., blocking or suppression) (6). Thus, this rhythm has been regarded as reflecting mental rest or cortical idling.

In the field of psychiatric rehabilitation, the EEG has been widely used as a simple indicator of brain activity, mainly for research purposes. Shiraiwa et al. reported the close correlation between frontal midline theta rhythm (Fmθ) (7) and autonomic nervous system function during handicraft activities (8). A systematic review also reported that EEG-based neurofeedback training was effective for patients with stroke and mental disorders (9–11). EEG has been applied not only in the field of psychiatric rehabilitation for mental disorders but also in the field of physical disorders.

Conventional EEG requires electrodes to be applied at several locations on the scalp according to the international 10–20 electrode placement method, which is time-consuming and laborious to apply, and the measurement is performed in a specific laboratory, which has disadvantages in terms of cost, simplicity, and restriction of the subject’s activity (12, 13). Until recently, access to EEG recording devices was limited to researchers and clinicians.

Recently, many companies have developed small, portable EEG amplifiers to meet research and clinical needs (14). These advancements allow EEG applications beyond the laboratory, overcoming the limitations of conventional devices. Wireless EEG technology has lowered the barrier for researchers in various fields, expanding its accessibility. However, commercialization has also led to exaggerated claims lacking scientific validation (14). Coates McCall et al. (2019) reviewed common claims about commercial wearable neurotech and found little supporting evidence for utility, safety, or efficacy (15).

In response to these trends, PGV Corporation introduced HARU-1, a sheet-type EEG device with high-precision sensors (16). Certified as a telemetric EEG device in Japan in 2020, HARU-1 is small, lightweight, and flexible, minimizing discomfort. It accurately measures EEG signals as low as 1 μV, operates wirelessly to reduce movement constraints and electromagnetic noise, and is cost-effective and non-invasive, facilitating broader EEG measurements. Unlike most commercial wearable EEG devices that use dry electrodes, HARU-1 employs conductive gel-based wet electrodes, which enhance adhesion and reduce contact impedance (13, 16, 17).

Previous studies using HARU-1 include a pilot study on dementia prediction (18), a sleep scoring system (19), and Fmθ detection during mental calculation (20). However, its application in EEG measurement during work activities remains limited, requiring further validation. This study aimed to assess HARU-1’s usefulness by comparing EEG results during eye opening and closing, considering its potential for psychiatric rehabilitation. Many wearable EEG validation studies have used the “Berger effect”—alpha waves appearing during eye closure and blocking upon eye opening—as an index (20–22). We hypothesized that HARU-1 would similarly detect alpha waves during resting eye closure, diminishing upon eye opening, and tested its effectiveness accordingly.

## 2 Methods

### 2.1 Subjects

The subjects were 24 young healthy subjects (mean age ± SD=23.8 ± 8.0) from the Department of Occupational Therapy, School of Comprehensive Rehabilitation Science, Faculty of Community Health, Osaka Prefecture University. They spoke Japanese as their first language and confirmed that they had no obvious neurological or psychiatric disorders, who were able to engage in their daily academic activities without difficulties. Before the experiment, the research was explained to the subjects orally and on paper, and informed consent was obtained. This study was conducted with the approval of the Ethics Committee of the Graduate School of Rehabilitation Science, Osaka Prefecture University (approval number: 2021-207). Twenty subjects (mean age ± SD = 24.5 ± 8.7) were analyzed by visual EEG reading by a specialist and supervising physician of the Japanese Society of Clinical Neurophysiology in the field of EEG, excluding four subjects who did not show alpha waves.

### 2.2 Equipment settings

**Figure 1:**
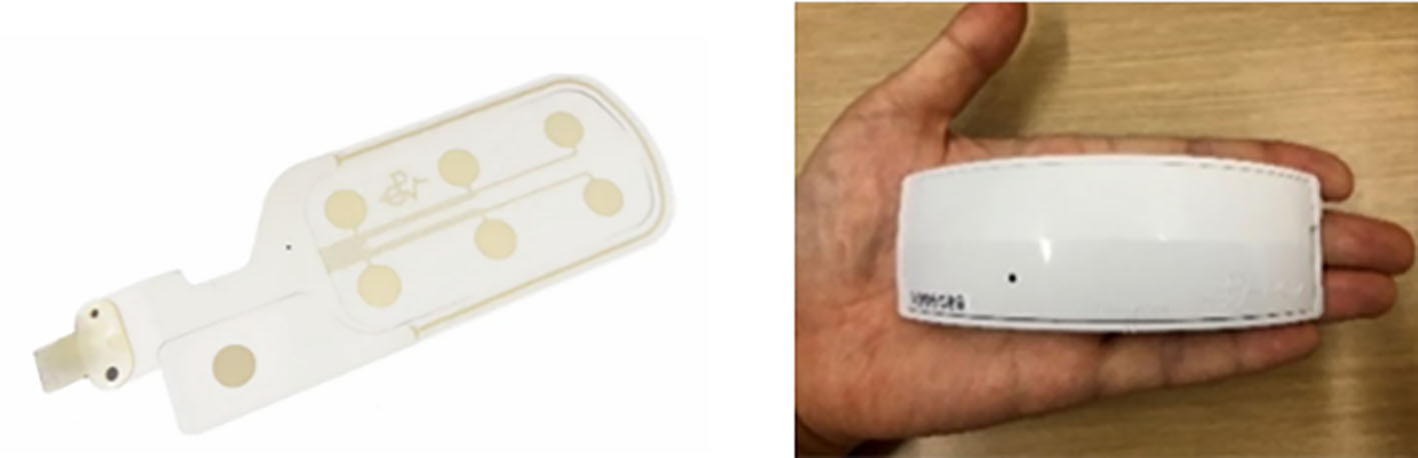
0-type EEG device HARU-1.

The HARU-1 is a multi-channel sheet-type EEG sensor that consists of a disposable sheet of electrodes and a wireless device (**Figure 1**). The wireless device contains an amplifier that receives signals from the electrodes, an AD converter and a battery. The electrode sheet is gentle to the human body, flexible and stretchable, and the wireless device is small and lightweight to reduce physical discomfort. EEG signals are transmitted via Bluetooth and recorded in a measurement application (Haru-Measure, PGV Corporation) on a measurement tablet (LAVIE Tab PC-TE507JAW, NEC). The reference electrode was placed on the mastoid process of the posterior part of the left auricle. The sampling rate was set at 250 Hz, and AC-generating devices were removed from the measurement environment to avoid environmental noise such as AC noise.

### 2.3 Experimental procedure

The subject’s forehead and left mastoid were wiped with ethanol cotton. A reference was placed on the left mastoid process and a sheet-type EEG device was attached to the forehead area. The subject then sat in a chair with a backrest while the EEG was attached and the measurement began. Subjects were instructed to keep their eyes closed in the chair-sitting position as the resting-eye-closed condition, and the EEG was measured for 2 minutes. For the resting-eye-open condition, the EEG was extracted from the part of the EEG measurement in which the eyes were not closed. (mean seconds ± SD = 28.8 ± 12.7). Although the protocols for the resting EEG measurement time differ in previous studies, the present study followed the protocol of a 2-minute closed-eye EEG measurement as the resting EEG measurement in a previous study of young healthy to middle-aged subjects21). To avoid evoked responses due to eye opening and closing21), we used data from 10 seconds after eye closure.

### 2.4 Pretreatment

The measured EEG signals were imported into signal processing software (BESA Research 6.0, BESA GmbH), and the High Pass Filter was set at 4.0 Hz, Low Pass Filter at 75.0 Hz, and Notch Filter at 60.0 Hz. The EEG (EEG) of each subject was visually checked by a specialist and instructor (in the field of EEG) of the Japanese Society of Clinical Neurophysiology, and areas where artifacts due to eye movements, electromyography, or body movements of 50 μV or higher persisted over a wide area were excluded.

### 2.5 Fast Fourier transform

After preprocessing, fast Fourier transform (FFT) was performed using BESA Research 6.0 for the reference electrode derivation method and the bipolar derivation method, and the power spectra within the θ wave (4.0-8.0 Hz), α wave (8.0-14.0 Hz), β wave (14.0-30.0 Hz) and γ wave (30.0-50.0Hz). The power spectrum within each band (4.0-8.0 Hz, 8-14.0 Hz, 14.0-30.0 Hz, and 30.0-50.0 Hz) was calculated. In the analysis, the time width was set to 1.02 seconds and the window to 256 points. Power values were compared using Mann-Whitney’s U test after checking for normality to confirm the significance of differences between eyes closed and eyes open for each EEG frequency band. Statistical analysis was performed using SPSS Statistics version 28.0.1.0 with a significance level of 0.050 to calculate the mean and statistical test volume.

### 2.6 Time-frequency analysis

For time-frequency analysis, after preprocessing, each subject’s EEG was epoxied for 2 seconds and artifact scans (Amplitude Threshold: 120μV) were performed using BESA Research 6.0. Time-frequency analysis was performed on each subject’s EEG with a frequency resolution of 0.25 Hz and a time resolution of 200 ms in the frequency range of 4.0-50.0 Hz for the reference electrode derivation method and the bipolar derivation method. Statistical processing software (BESA Statistics 1.0, BESA GmbH) was used to perform group statistics on the results of time-frequency analysis for all subjects using a permutation test based on a t-test with a correspondence between the two conditions (closed eye - open eye). The significance level was set at 0.050. BESA Statistics was set to Average over Time mode to average the 2-second time windows. The statistical software used in this study addressed the issue of multiple comparisons by conducting a preliminary t-test to investigate significant differences between the two conditions, followed by a permutation test on the data obtained from the preliminary test statistics (23).

## 3 Results

### 3.1 Fast Fourier transform in the reference electrode derivation method

**Figure 2:**
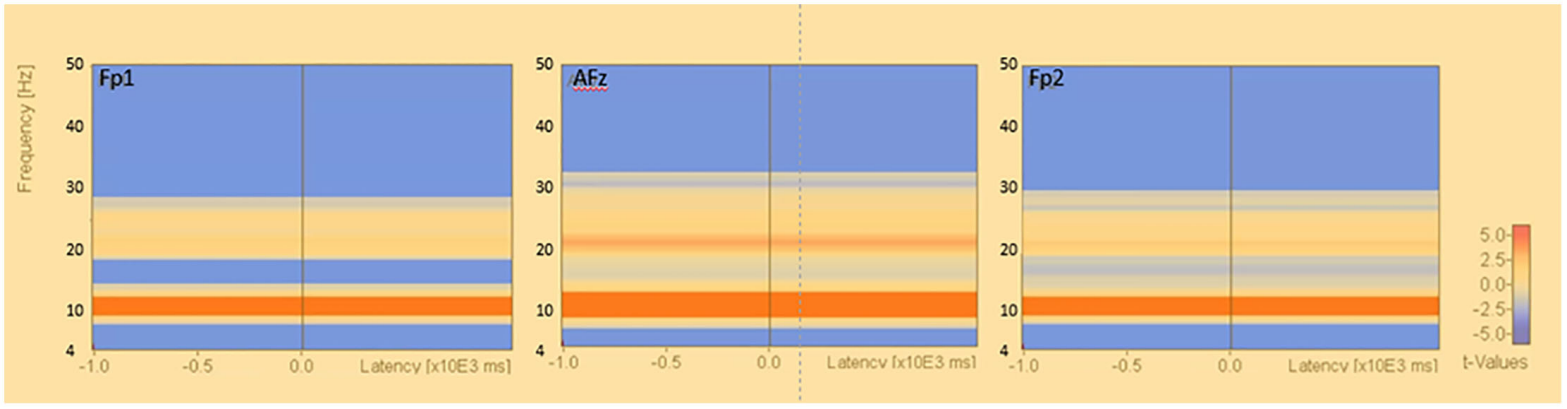
Fast Fourier transform under eyes-closed condition and eyes-open condition (reference electrode derivation). Fast Fourier Transforms are performed in the eyes closed and open conditions, and power values are compared by Mann-Whitney-U test in theta, alpha, beta, and gamma conditions, respectively. The electrodes are AFz, Fp1, and Fp2. **Figure 3**. Fast Fourier transform under eyes-closed condition and eyes-open condition (bipolar derivation). Fast Fourier Transforms are performed in the closed-eye and open-eye conditions, and power values are compared by Mann-Whitney-U test in theta, alpha, beta, and gamma, respectively. The electrodes are Fp1-AFz, AFz-Fp2, and Fp2-Fp1.

The power spectra for each frequency band resulting from Fast Fourier transform using the reference electrode derivation method are shown. In the closed eye condition, the alpha power of AFz (U=-3.771, p<0.001), Fp1 (U=-3.771, p<0.001), and Fp2 (U=-3.733, p<0.001) were significantly increased compared to the open eye condition. In the open eye condition, γ power of AFz (U=2.8, p=0.005), Fp1 (U=3.547, p<0.001) and Fp2 (U=3.136, p=0.002) increased significantly. no significant difference appeared in β and θ power (**Figure 2**).

### 3.2 Fast Fourier transform in the bipolar derivation method

**Figure 3:**
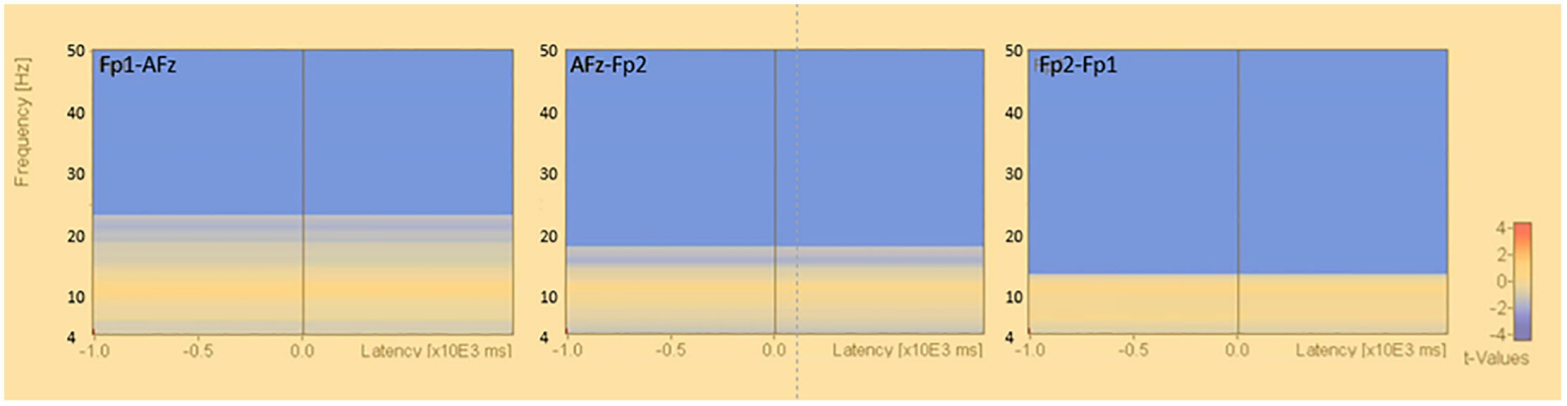
Fast Fourier transform under eyes-closed condition and eyes-open condition (bipolar derivation). Fast Fourier Transforms are performed in the closed-eye and open-eye conditions, and power values are compared by Mann-Whitney-U test in theta, alpha, beta, and gamma, respectively. The electrodes are Fp1-AFz, AFz-Fp2, and Fp2-Fp1.

The results of the fast Fourier transform in the bipolar derivation method showed that, in the open eye condition, the θ power of AFz-Fp2 (U=2.073, p=0.038) and the α power of AFz-Fp2 (U=2.036, p=0.042), beta power for Fp2-Fp1 (U=3.808, p<0.001), AFz-Fp2 (U =3.920, p<0.001), Fp1-AFz (U=3.510, p<0.001), and γ power for Fp1-AFz (U=3.808, p<0.001), AFz-Fp2 (U=3.743, p<0.001), Fp2-Fp1 (U=3.696, p<0.001) significantly increased (**Figure 3**).

### 3.3 Population statistics using the reference electrode derivation method

**Figure 4:**
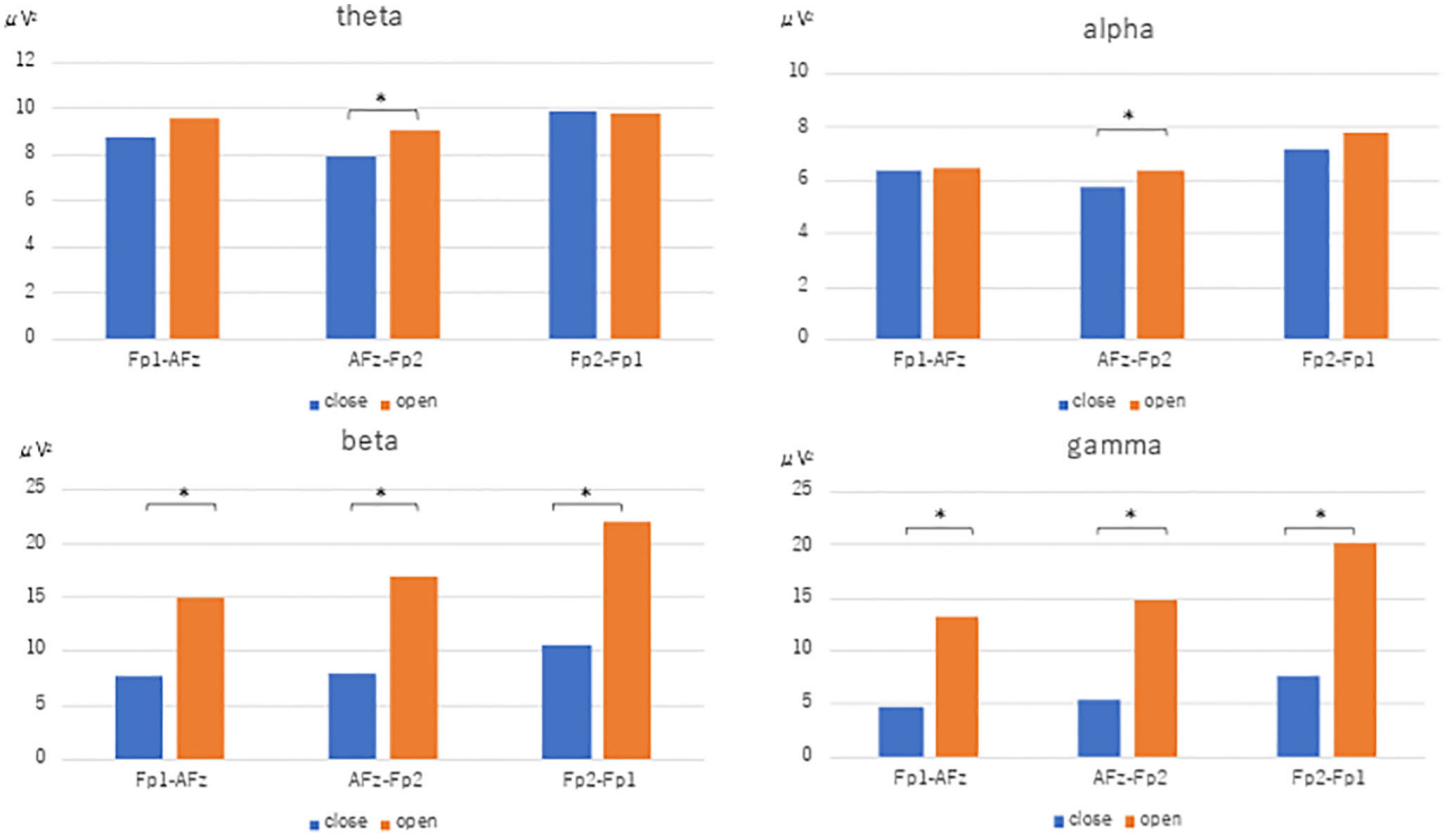
Time-frequency analysis (reference electrode derivation). Results of permutation tests based on the corresponding t-tests of the results of the time-frequency analysis in the reference electrode derivation (eyes closed - eyes open). **Figure 4** Time-frequency analysis (bipolar derivation). Results of permutation tests based on the corresponding t-tests for the results of time-frequency analysis in the bipolar derivation (eyes closed - eyes open), *: p<.0.05.

The results of the group statistics by permutation test in the reference electrode derivation method showed that there was a significant increase in activity in the alpha band at approximately 8.0-13.0 Hz in the resting closed-eye condition compared to the resting open-eye condition (Fp1: p<0.034, Fp2: p<0.039, AFz: p<0.009). There was also a decrease in the theta band at approximately 4.0-7.9 Hz (Fp1: p<0.005, Fp2: p<0.005, AFz: p<0.017) and a significant decrease in activity in the beta and gamma bands at approximately 27.0-50.0 Hz (Fp1: p<0.036, Fp2: p<0.001, AFz: p<0.001) (**Figure 4**).

### 3.4 Population statistics using the bipolar derivation method

**Figure 5:**
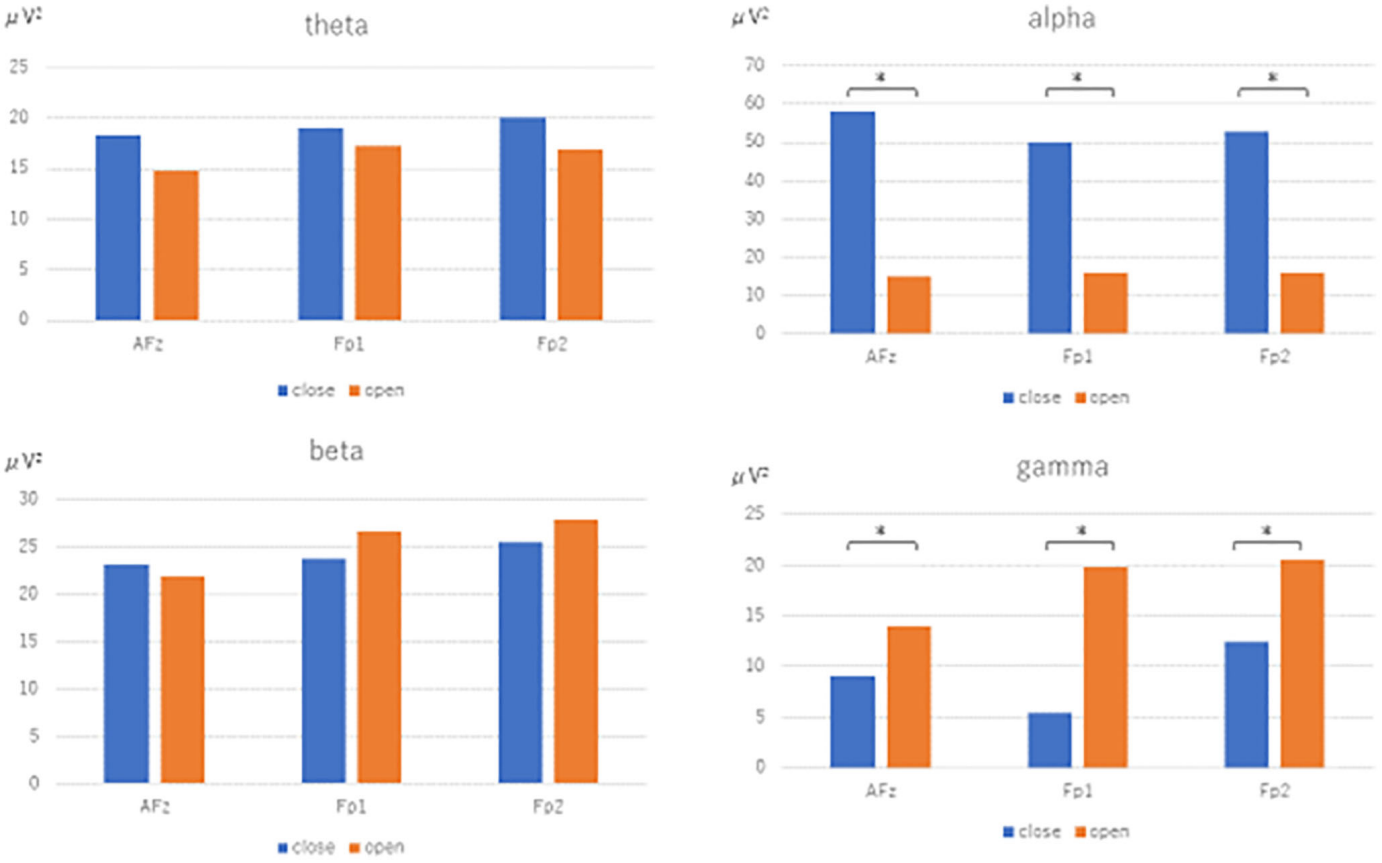
Time-frequency analysis (bipolar derivation). Results of permutation tests based on the corresponding t-tests for the results of time-frequency analysis in the bipolar derivation (eyes closed - eyes open), *: p<.0.05.

The results of the population statistics by permutation test in the bipolar derivation method showed that the β and γ bands were decreased in the resting closed-eye condition in the Fp1-AFz, Fp2-Fp1, and AFz-Fp2 regions at approximately 13.0-50.0 Hz (Fp1-AFz: p<0.012, Fp2-Fp1: p<0.002, AFz-Fp2: p<0.004) (**Figure 5**). No significant changes in activity were observed in the α and θ bands.

## 4 Discussion

In this study, we examined the usefulness of a sheet-type EEG device in healthy young subjects, using the appearance of alpha waves during eye closure and the blocking of alpha waves during eye opening as indices. The results of FFT (**Figure 2**) and population statistics of time-frequency analysis (**Figure 4**) in the reference electrode derivation method showed that the activity of the α band increased during rest-wake eye closure, while the activity of the α band decreased during resting-state eye opening, and the activity of the θ band, β and γ bands increased. On the other hand, the results of the population statistics of the fast Fourier transform (**Figure 3**) and time-frequency analysis in the bipolar derivation method (**Figure 5**) showed no significant difference in all the α-bands, and the significant increase in activity in the β and γ-bands during eye opening was still observed. The weaker significance observed in the bipolar derivation method may be attributed to a cancellation effect, where differences in electrode placement reduce signal amplitude. This could lead to diminished detection of alpha wave modulation compared to the reference electrode method. Among the derivation methods used, the reference electrode method demonstrated clearer alpha wave modulation than the bipolar derivation method. While a direct statistical comparison was not performed, the reference electrode method may be preferable when aiming to detect alpha wave changes robustly. However, each derivation approach has its advantages depending on the research context, and further studies are needed to determine the optimal method for specific applications. In conclusion, the sheet-type EEG device attached to the forehead confirmed the appearance of α waves during eye closure and the blocking of α waves by eye opening, demonstrating the usefulness of the sheet-type EEG device.

The “Berger effect” is well-known EEG phenomenon as an increase in alpha band activity during eye closure and a decrease in alpha band activity during eye opening (1, 2). Legewie compared EEG during the task and resting EEG in both open and closed eye conditions (2). Gao et al. used the increase or decrease in alpha band activity during eye opening and closing as an index to verify the usefulness of wearable EEG devices (21). The result in the present study that “alpha band activity increased more in the closed eye condition than in the open eye condition” is consistent with previous studies (21, 22). This suggests that the sheet-type EEG captures alpha waves as accurately as conventional EEGs. Since the HARU-1 uses wet electrodes made of conductive gel (16), this confirms that the HARU-1 has the characteristic of lower impedance than the dry electrode because of the wet electrode.

In the present study, we found β-band activity in the Fp1 band of approximately 14.0-19.0 Hz. Previous studies have shown that theta and beta bandwidths are increased in Fp1 and Fp2 during eye opening (23, 24). A study by Barry et al. showed that beta waves were significantly larger in the left frontal hemisphere in older subjects than in younger subjects under open-eye conditions. Since two middle-aged subjects were included among the healthy subjects, aging may be a contributing factor (25). γ waves are high-frequency components exceeding 30 Hz, and have been identified since the development of digital EEGs. However, γ-band activity is often mixed with artifacts such as EMG and ocular muscle activity around the head and neck, so it is necessary to carefully distinguish γ waves from EEG signals (26).

In the present study, alpha band activity appeared in the forehead region when the eyes were closed using the reference electrode method, but no activity was observed using the bipolar derivation method. One reason for this may be that the alpha waves in the mastoid process of the left ear, which was the location of the reference electrode, were captured, since alpha waves are predominantly generated in the occipital region (27).

Sleep EEG studies have reported that alpha waves and other frequency components indicate the arousal state (27), and are applied to BIS monitors that objectively measure the degree of anesthesia-induced sedation (28–30). The Glasgow Coma Scale (GCS) and Japan Coma Scale (JCS), which are widely used in anesthesia, are evaluation methods to grasp the level of consciousness of patients, but they require knowledge and skills of medical personnel, and the same score is said to have different degrees of symptoms. On the other hand, the sheet-type EEG device can read the EEG in real time and objectively determine the patient’s level of arousal. In psychiatric rehabilitation, the sheet-type EEG device suggests the possibility of conducting evaluation and intervention while monitoring the patient’s arousal level while wearing the sheet-type EEG device.

As future applications based on the findings and methodology outlined in this study, the use of HARU-1 represents a step towards wearable and non-invasive EEG monitoring. Future applications could involve integrating this technology into everyday devices like smartwatches or headbands for continuous monitoring of brain activity in various settings, such as during sleep, meditation, or while performing cognitive tasks. HARU-1 also could be employed for health monitoring purposes, especially in assessing neurological conditions or mental health disorders. By analyzing changes in brainwave patterns, it could provide valuable insights into conditions like epilepsy, attention-deficit/hyperactivity disorder (ADHD), or anxiety disorders.

The real-time monitoring capabilities of HARU-1 could be utilized in biofeedback therapy sessions. Patients could receive immediate feedback on their brain activity, helping them learn to regulate their mental states and emotions. This could be particularly beneficial for stress management, relaxation techniques. HARU-1 could be incorporated into neurofeedback training programs, allowing users to visualize their brainwave patterns in real-time and learn to modulate them through mental exercises or relaxation techniques.

This sheet-type EEG device HARU-1 is small and comfortable to wear, and can be used for long periods of time for physical activity, making them suitable for a wide range of subjects, from the elderly to infants. Most of the conventional work studies using EEGs have focused on small movements, such as button-pushing tasks, to limit movement. Since the work activities that humans perform daily are complex and continuous movements, it is thought that this device can capture brain activity in more natural movements. In the future, we would like to increase the number of subjects and measure and analyze EEG during more complex tasks to establish its usefulness as an objective index in the field of psychiatric rehabilitation.

Limitations of the sheet-type EEG itself are that the number of electrodes is limited to three channels in the forehead area, that it is prone to artifacts caused by eye movements, and that the spatial resolution is low. In addition, there are several other limitations that should be acknowledged: 1. The study’s sample size is relatively small, with only 24 young healthy subjects. Additionally, the subjects were recruited from a specific department of a university, potentially limiting the generalizability of the results to other populations. The health status of the participants was based solely on self-reported information without any objective neurological or psychiatric evaluations. This reliance on self-reports may have introduced biases or inaccuracies in the inclusion criteria. 2. The use of a 2-minute EEG recording for the resting state may not capture sufficient data for robust analysis, particularly considering individual variability in EEG patterns. The decision to use data from 10 seconds after eye closure to avoid evoked responses is arbitrary and lacks clear justification. However, the results of the present study confirmed that the sheet-type EEG device can be used to measure alpha waves while avoiding the complexity of conventional EEG measurement.

In conclusion, our study investigated the efficacy of the HARU-1 sheet-type EEG device in discerning alpha wave patterns during rest with closed eyes and their attenuation upon eye opening. The findings substantiate our hypothesis that alpha waves would manifest during resting eye closure, akin to conventional EEG devices, and would diminish upon eye opening, validating the utility of HARU-1. These outcomes collectively affirm the ability of the sheet-type EEG device to capture alpha wave dynamics during resting conditions and their modulation with eye states. Consequently, the HARU-1 device emerges as a promising tool for non-invasive EEG monitoring, offering insights into neural activity patterns with potential applications in various research and clinical settings.

## Data Availability

The raw data supporting the conclusions of this article will be made available by the authors, without undue reservation.
